# Game Theory for Wireless Sensor Networks: A Survey

**DOI:** 10.3390/s120709055

**Published:** 2012-07-02

**Authors:** Hai-Yan Shi, Wan-Liang Wang, Ngai-Ming Kwok, Sheng-Yong Chen

**Affiliations:** 1 College of Computer Science and Technology, Zhejiang University of Technology, Hangzhou 310023, China; E-Mail: csshy@usx.edu.cn; 2 School of Computer Science and Technology, Shaoxing University, Shaoxing 312000, China; 3 School of Mechanical and Manufacturing Engineering, The University of New South Wales, Sydney, NSW 2052, Australia; E-Mail: nmkwok@unsw.edu.au

**Keywords:** wireless sensor network, game theory, scheduling, optimization, mechanism

## Abstract

Game theory (GT) is a mathematical method that describes the phenomenon of conflict and cooperation between intelligent rational decision-makers. In particular, the theory has been proven very useful in the design of wireless sensor networks (WSNs). This article surveys the recent developments and findings of GT, its applications in WSNs, and provides the community a general view of this vibrant research area. We first introduce the typical formulation of GT in the WSN application domain. The roles of GT are described that include routing protocol design, topology control, power control and energy saving, packet forwarding, data collection, spectrum allocation, bandwidth allocation, quality of service control, coverage optimization, WSN security, and other sensor management tasks. Then, three variations of game theory are described, namely, the cooperative, non-cooperative, and repeated schemes. Finally, existing problems and future trends are identified for researchers and engineers in the field.

## 1. Introduction

### 1.1. Wireless Sensor Networks

A wireless sensor network (WSN) is a network of thousands of resource-constrained sensors whose communications with a central station are conveyed by means of wireless signals. A sensor node is generally comprised of four basic elements, including a sensing unit, a processing unit, a transceiver unit, and a power unit. The WSN is frequently deployed for sensing the area of interest where data captured encompass light, pressure, sound, and others. Sensor nodes in WSN mainly use a broadcast communication paradigm where the sensor signals are used in further analyses of the sensed environment. WSN is preferred as the sensor system architecture with regard to its inherent redundancy but is susceptible to disadvantages caused by limited operation life-time. Differ from other wired networks, the use of WSNs are usually restricted by energy stored, computation capability, memory, plethoric information flow, and short communication distance [1]. Since the sensor nodes are often densely deployed in a sensing field, it is difficult and costly to replace faulty sensor nodes manually. Furthermore, sensor nodes may have no global information of the whole network and the topology of a WSN varies frequently [2,3].

### 1.2. Application Examples

With the high degree of deployment flexibility, applications of WSN are vast and can be broadly classified into the monitoring and tracking categories. Monitoring applications include environmental monitoring such as forest fire detection, biocomplexity mapping of the environment, flood detection, precision agriculture; health monitoring contains tele-monitoring of human physiological data, monitoring doctors and patients conditions and drug administration in hospitals; inventory location monitoring; factory, machine, chemical and structural monitoring. Military monitoring examples can be found in monitoring friendly forces, equipment and ammunition, battlefield and terrain surveillance, reconnaissance of opposing forces, targeting, battle damage assessment, nuclear, biological and chemical attack detection. Tracking applications include objects, animals, humans, vehicles, and military enemy tracking. These applications are made possible due to the fact that WSN has a short system setup time and sensors can be disposed with acceptable operation cost.

### 1.3. Need for Game Theory

The flexibility, fault tolerance, high sensing fidelity, low-cost and rapid deployment characteristics of WSNs are desirable features in creating many new and exciting application areas for remote sensing, detecting, tracking, and monitoring. However, it is non-trivial and very involved to design an optimal WSN to satisfy performance objectives such as maximum sensing coverage and extended operation periods. In order to obtain a practical and feasible WSN and due to the operation nature of the network, game theory (GT) is regarded as an attractive and suitable basis to accomplish the design goal. Game theory is a branch of mathematics and can be used to analyze system operations in decentralized and self-organizing networks. GT describes the behavior of players in a game. Players may be either cooperate or non-cooperative while striving to maximize their outcomes from the game. In this regard, sensors manage their operations in terms of power resources devoted to sensing and communicating among themselves and with a global controller such that the assigned task could be completed effectively as desired [4].

### 1.4. Motivation

With the rapid development in electronics and wireless technology, WSN will certainly find more and more application when the need for environment sensing arises. On the other hand, the developments of WSN theory and systems have received a lot of attention in both the industry sector and research community. Among many alternative approaches, GT has been increasing applied in the design of WSNs, thus, the scope of this paper is restricted to the use of GT for WSNs.

**Figure 1.**
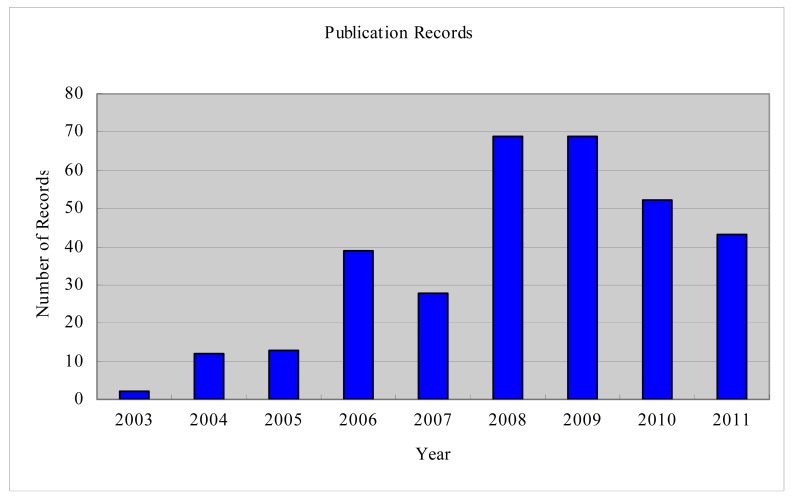
Yearly publications on GT for WSN.

From 2003 to 2011, about 330 research papers with topics on or closely related to GT for WSN were published. Figure 1 shows the yearly distribution of these published papers. The number of records for 2011 is not complete because some publications have not been included in the indexing databases. A relatively smaller portion of the contributions in this area has been summarized in [5,6]. Machado and Tekinay [5] reviewed 29 publications which mainly focusing on the use of game-theoretic approaches to formulate problems related to security and energy efficiency. Shen *et al.* [6] summarized 30 publications of the existing game theoretical approaches that are designed to strengthen WSN security.

Among the variety of developed methods using GT, the main differences and remarkable features can be briefly summarized below. Cooperative game theory provides analytical tools to study the behavior of rational players when they cooperate and consider the utility of all the players [7,8]. Non-cooperative game theory also covers a broad range of applications in WSN [9,10]. In non-cooperative game theory, the nodes buy, sell, and consumer goods in response to the prices that are exhibited in a virtual market. A node attempts to maximize its profit for taking a series of actions. Whether or not a node receives a profit is decided by the success of the action. Note that non-cooperative game theory is mainly focused on each user's individual utility rather than the utility of the whole network. On the contrary, cooperative game theory can achieve general pareto-optimal performance and maximize the entire network's payoff while maintaining fairness. In addition to cooperative and non-cooperative game theories, repeated game theory is concerned with a class of dynamic games, in which a game is played for numerous times and the players can observe the outcome of the previous game before attending the next repetition [11]. The commonly used GT methods for solving WSN problems are listed in Table 1.

### 1.5. Organization of the Survey

Our aim is to provide a better understanding of the current research issues in this field with overviews of the main ideas and the basic game types of various approaches. This survey concentrates on the contributions of the past 10 years. It will provide researchers with a better understanding of game-theoretic solutions for WSNs and further research trends are identified. We propose and hope more and more researchers all over the world will be encouraged to join this vibrant research area.

The rest of this paper is organized as follows: Section 2 briefly describes three kinds of GT for WSNs and how these schemes are used to solve WSN design problems. Section 3 lists the applications of GT in WSNs. We focus on the techniques available in WSN implementations using GT and show the available methods and solutions to specific applications. Section 4 is a discussion of our impression on current and future trends. A conclusion is drawn in Section 5.

## 2. Typical Categories of Game Theory

### 2.1. Common Notations

There exist several main terminologies in GT and they are listed in Table 2 below before a description of the formulations is given:

### 2.2. Basics of Game Theory

Game theory is increasingly attracting more attention as a mechanism to solve various problems in WSNs [71–76]. Generally, a game consists of a set of players, a set of strategies for each player and a set of corresponding utility functions. A norm form game of a WSN of *n* sensor nodes is given by a 3-tuple G = <*N, S, U*>. Here, *G* is a particular game, where *N* = {*n*_1_, *n*_2_, …, *n*_n_} is a finite set of the sensor nodes. *S* = {*S*_1_, *S*_2_, …, *S*_n_}, is the strategy space of the sensor node *i* can select from is represented by *S*_i_ (*i* = 1, 2, …, *n*). *U* = {*u*_1_, *u*_2_, …, *u*_n_} is the corresponding payoff function of node *i* represented by *u*_i_ (*i* = 1, 2, …, *n*), *u*_i_ is a utility value of each node receives at the end of an action.

A strategy for a player is a complete plan of actions in all possible situations in the game. The players try to act selfishly to maximize their consequences according to their preferences. We have to formulate the payoff functions in a way that will help node *i* to select a strategy *S*_i_ that represents the best response to the strategies selected by the other *n*-1 nodes. Here, *s*_i_ is the particular strategy chosen by node i and **s**_-i_ is the particular strategies chosen by all of the other nodes in the game. For strategies **s** = {*s*_i_, **s**_−i_}, it is called a strategy profile or sometimes a strategy combination. Every different combination of individual choices of strategies can produce a different strategy profile. The strategy profile ***s*** = {*s*_1_, *s*_2_, …, *s*_n_ ∣ *s*_i_ ∈ *S*_i_, *i* = 1, 2, …, *n*} needs to place the nodes responding to a Nash Equilibrium (NE). It is a solution concept that describes a steady state condition of a game involving two or more players, in which each player is assumed to know the equilibrium strategies of the other players, and no player has anything to gain by changing only its own strategy unilaterally. NE is identified wherein no nodes will rationally choose to deviate from his chosen strategy otherwise it will diminish its utility, *i.e., u*_i_ (*s*_i_, **s**_-i_) ≥ *u*_i_ (*s*_i_*, **s**_-i_) for all *s*_i_* ∈ *S*_i_.

A utility function describing player preferences for a given player assigns a number for every possible outcome of the game with the property that a higher number implies that the outcome is more preferred. In literature [59], the utility is defined as:

(1) $$u_{i}(s_{i},s_{-i})=\frac{br}{Fs_{i}}f(\gamma_{j})$$

where *u*_i_ (*s*_i_, **s**_−i_) can be considered as the utility of node *i* transmitting information to a node *j, b* is the number of information bits in a packet of size *F* bits, *r* is the transmission rate in bits/sec using strategy *s*_i_, and *f* (*γ_j_*) is efficiency function which increase with expected SINR of the receiving node. The efficiency function is defined as:

(2) $$f(\gamma_{j})={\left(1-2P_{e}\right)}^{F}$$

where *P*_e_ is the bit error rate which depends on the channel state and interference from other nodes.

Schillings and Yang [77] used the GT framework to construct a query-based Versatile Game Theoretic Routing Protocol (VGTR) to accomplish the extraction of data from WSN. Three payoff functions are used. The first two represent node survivability and the third one represents the importance of the information collected. Node survivability represents the capability of a node to remain in contact with the Sink for as long as possible. To explain these three payoff functions clearly, several definitions were used, such as Upstream Potential Path Nodes (UPPN) Robustness, and Neighbor Robustness.

After several years of development, there are several variations of game theory applicable in wireless sensor networks. The developments include cooperative game theory [8], non-cooperative game theory [9,10], and repeated game theory [11].

### 2.3. Cooperative Game Theory

#### 2.3.1. Basics of Cooperative Game Theory

To reduce the whole WSN's energy consumption and prolong its lifetime, some nodes will cooperate and form a coalition. Coalitional game theory is one of the most important cooperative game theory, thus, cooperative game theory is sometimes denoted as coalitional game theory [7]. For a WSN obeying the cooperative game theory, cooperating groups are formed and players choose strategies to maximize their own groups' utility. Coalitional game theory allows a reduction of power consumption in WSN by forming coalitions.

Saad *et al.* [7] proposed a merger and split approach for coalition formation, which calculates the value of the utility function for every possible permutation of nodes and finds groups with the best utility value. Here, grouping is treated as a basic method to organize sensor nodes for cooperation between nodes. In this formation, the nodes know nothing about the grouping. On the other hand, a group leader is assigned as a special node which processes the information of the newly entered sensor nodes and decides who will be their possible group member in a group.

We can group the nodes in two ways for different applications: (1) All the sensor nodes have similar sensed data could be placed in the same group, for example, sensing application. (2) The sensor nodes with shorter distances between them are allocated in the same group, for example, sending data from a source node to the sink. Apt and Witzel [78] proposed a generic approach for coalition formation through simple merge and split operations.

Cooperative game theory can be further categorized into two branches: Transferable-utility game (TU) [30] and non-transferable-utility game (NTU) [31]. In TU game the payoff of the measurement allocation game is transferable. In NTU game the payoff for each agent in a coalition depends only on the actions selected by the agents in the coalition.

##### 2.3.1.1. Shapley Value

Shapley value is derived from the solution concept in cooperative game theory which was defined by Shapley [48]. It is one of the most important solution concepts in cooperative game theory and a representative single-valued solution concept in the theory of cooperative games [30]. An agent's Shapley value gives an indication of its prospects of playing the game in cooperative game theory. It is useful when there is a need to allocate the worth that a set of players can achieve if they agree to cooperate. The Shapley value was defined for TU games and NTU games in regard to conflicts among players [79].

Wu *et al.* [80] adopted Shapley values as the coalition's payoff sharing mechanism based on game theory. Byun *et al.* [81] investigated the spatial correlation in the context of measurement allocation that enables efficient detection of phenomena in inaccessible area. Byun and Balasingham [82] modeled the measurement allocation problem into a cooperative game using the Shapley value. They found a set of stable payoff allocations such as core values for all agents to satisfy. In order to avoid intractability in the computation of exact Shapley value, they deployed a randomized method to compute the approximate Shapley value within a reasonable time. Fatima *et al.* [83] proved that finding the exact Shapley value is Sharp-P-complete (#P- complete). Castro *et al.* [84] developed an approximation technique to compute the Shapley value based on the randomized method for cooperative game. They used sampling to estimate the Shapley value and any semi-values.

##### 2.3.1.2. Coalition Formation

Agastya [85] studied a dynamic social learning model by focusing on allocations and completely abstracts from coalition formation process. Wu *et al.* [86] formed the WSN coalitions on the basis of Markov-process, and proposed the concept of absorption coefficient to measure the coalitional profiles and then use NE to determine the approximate data transfer strategies of the formed coalitions. Some nodes in a WSN form a coalition by transferring data coordinately instead of transferring independently in order to reduce energy consumption. Wu *et al.* [87] focused on how to select a proper transmission scheme for improving the energy efficiency. They modeled the transmission scheme selection problem as a non-transferable coalition formation game with the characteristic function based on the network lifetime. They further proposed a simple algorithm based on a merge-and-split rule and the Pareto order to form coalition groups among individual sensor nodes.

Gharehshiran and Krishnamurthy [49–51] used cooperative game theory as a tool to devise a distributed dynamic coalition formation algorithm in which nodes autonomously decide which coalition to join while maximizing their feasible sleep times. The sleep time allocation problem is formulated as a non-convex cooperative game and the concept of the core is exploited to solve this problem. They solved two problems: (1) What are the optimal coalition structures for localizing multiple targets with a pre-specified accuracy? (2) How can nodes dynamically form optimal coalitions to ensure that the average sleep time allocated to the nodes is maximized? The two questions are solved nicely within the framework of coalition formation in a cooperative game.

**Figure 2.**
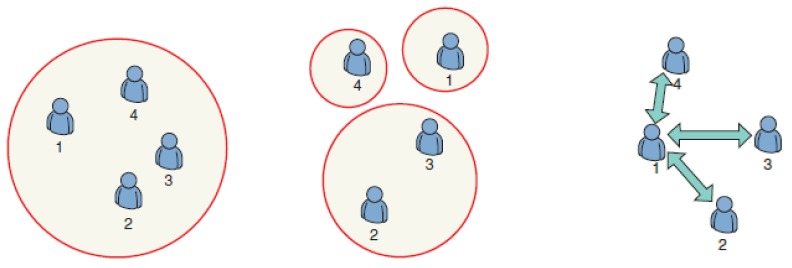
Classification of coalitional games: Class I, II, and III [7].

Saad *et al.* [7] classified coalitional game theory into three categories: Class I—canonical coalitional games (as a coalition comprising all nodes, it is not optimal), Class II—coalitional formation games (as a main branch of cooperative game theory, comprises several disjoint coalitions), and Class III—coalitional graph games (Figure 2). The classification is intended to provide an application-oriented approach to coalitional game theory.

According to the coalitional game theory, *n* sensor nodes form the game by a triplet G = <*N, v, S* > characteristic form, where *G* is a coalitional game, *N* = {*1*, …, *N*} is a fixed set of players called the grand coalition (the coalition comprising all nodes, it is not optimal), *S* = {*S*_1_, …, *S_l_*} denotes a coalitional structure and a partition of *N*, and *v* is a characteristic function of coalition value which quantifies the worth of a coalition in a game. The coalitional game *G* seeks to form cooperative coalitions to strengthen their positions in the game. Any coalition *S_i_*(*i* = 1,…, *l*) represents an agreement between the players in *S* to act as a single entity. Nodes in each coalition share measurements to localize a particular target.

#### 2.3.2. Communication, Spectrum Allocation and Routing

For instance, Ma *et al.* [42] proposed a cooperative strategy for multi-user symmetrical cooperative communication networks. An optimal bandwidth allocation framework is suggested that utilizes NBS from cooperative game theory. Roosta *et al.* [88] examined the robust detection and estimation problem using recent results for cooperative sensing in cognitive radios and multi-object tracking in sensor networks. The authors of literature [88] considered different types of lying behavior: (1) The liar's behaviors remain constant in the simplistic cases. (2) The lying behaviors of the users change over time in more complex cases. Sang-Seon and Balasingham [89] proposed a coalitional game theoretic approach to the power control problem in resource constrained WSNs and model the problem as two-sided one-to-one matching game and deploy deferred acceptance procedure that produces a single matching in the core. Moreover, as the procedure iterates repeatedly, a certain stable state is achieved where no sensor can anticipate improvements in their power efficiency as far as all of them are subject to their own QoS constraints.

Vanbien *et al.* [90] had designed a centralized dynamic spectrum allocation scheme to enhance the spectrum utilization and maximize the profit of operators for cooperative wireless networks. In this scheme, the economic factor of the spectrum of wireless systems is considered in order to guarantee the fairness for the spectrum allocation. Radio access technologies (RATs) are considered as cooperative players participating in cooperative games for spectrum allocation. As an attractive solution for n-person cooperative game with transferable utility, the Shapley value is adopted to handle the profit allocation among RATs. Kazemeyni *et al.* [91] modified the *Ad-Hoc* On-demand Distance Vector (AODV) routing protocol [13] using coalitional game theory. AODV is a reactive protocol where the route is created when needed.

As to the collaborative technique employed in sensor networks, an integrated multi-target collaborative tracking model was constructed based on mobile dynamic coalition under the background of multi-satellite tracking [92]. Sergi and Vitetta [93] used game theory to set up a cluster of cooperative nodes in WSN. A source node gives his sampling data to a destination node needing other nodes' relay. There are two scenarios for the behavior of relay nodes. In the first scenario all the nodes are all prone to cooperation, while in the second case they are all selfish. In the first scenario a node will make its choices without expecting his personal reward if he can bring benefit to the network by his actions. In the second scenario proper polices are needed for cooperation enforcement. A node acquires some credits which represented by virtual money or a reputation level when it cooperates with other nodes. Moreover, we can use non-cooperative game theory in the second scenario for the selfish nodes.

### 2.4. Non-Cooperative Game Theory

Non-cooperative game theory studies strategies between interactions among competing players. In the game, a player is called an agent and his goal is to maximize its utility by choosing its strategy individually, in other words, each player is selfish but rational in a non-cooperative game. Non-cooperative game theory uses a utility function to find the NE [61–66]. Non-cooperative game theory is mainly applied in distributed resource allocation, congestion control, power control, spectrum sharing in cognitive radio and many others. With the concepts from economics and game theory, Wu and Wei [52] proposed a mechanism design to handle incentives of strategic agents. A power control model based on non-cooperative game theory is given in [94].

#### 2.4.1. Applications in WSN

Conventional centralized information fusion and control architectures will be challenged by developments in sensor networks that allow sophisticated autonomous sensors, owned by different stakeholders with individual goals, to interact and share information. Rogers *et al.* [95] advocate the use of tools and techniques from computational mechanism design (CMD), a field at the intersection of computer science and non-cooperative game theory, to address the challenges posed by these networks. Shamik *et al.* [59] formulated a non-cooperative game under incomplete information for the distributed sensor nodes. The benefit and the cost were defined the existence of NE was investigated. A metric called distortion factor was formulated to assess the performance of such system and compare it with systems that would allow any continuous power levels. “Cognitive radio” is an emerging technique to improve the utilization of radio frequency spectrum in wireless networks. In [96], Niyato and Hossain considered the problem of spectrum sharing among a primary user and multiple secondary users. They formulated this problem as an oligopoly market competition and used a non-cooperative game to obtain the spectrum allocation for secondary users. Haksub *et al.* [97] proposed a non-cooperative game based energy efficient MAC algorithm which makes the sensor nodes consume their energy efficiently.

#### 2.4.2. Dynamic Communication Assignment

Stankovicacute *et al.* [98] considered the problem of distributed convergence to a NE based on minimal information about the underlying non-cooperative game. Interference problems for spectrum-agile networks were addressed by allowing the networks to dynamically change channel in literature [99]. For insight into dynamic channel-change strategies, authors modeled the networks as autonomous players in a multistage non-cooperative game-theoretic model. Here the networks are assumed to be highly interfering, *i.e.*, when two or more networks exist on a single channel they cannot successfully carry traffic. Each network seeks to minimize its time to find a clear channel. The game-theoretic analysis reflects the motivations and choices of independent, rational, selfish decision makers that do not trust one another. They analyzed appropriate game-theoretic solutions in an un-trusted environment, and compare results with socially optimal decisions that would maximize the expected benefit of all coexisting networks in a trusted environment.

Sensor energy is limited, and the sensor is selfish but rational in WSN. If a node cannot get some profits, it will not perform the task. Of course, there will be some nodes that may exaggerate their real capacities to get some more tasks for profits. These dishonest or selfish behaviors will seriously affect the efficiency of WSN. Therefore, incentives should be provided to force nodes to obey the prescribed algorithms and report truthfully their capacity. Mechanism design in GT can resolve this problem [55].

#### 2.4.3. Mechanism Design

Mechanism design not only provides the right incentives, but also to ensure the participants tell the truth. It can balance individual interests and common interests. The following terms related to mechanism:

**Definition 1 (Mechanism)** Mechanism can be expressed as *M* = (*λ, P*), where *M* means some kind of mechanism, *λ* is the output function, *λ* = *λ* (*λ*_1_, *λ*_2_, …,*λ*_n_), *P* is the payment function, *P* = *P* (*P*_1_ (*λ*), *P*_2_ (*λ*), …, *P*_n_ (*λ*)).
**Definition 2 (Strategyproof Mechanism)** In the mechanism *M*, any agent *i*, its true value *t_i_*, the bid vector ***b***_−*i*_, agent *i* in order to obtain maximum profit only by submitting a real bid (*i.e., b_i_* = *t_i_*), the mechanism is strategyproof.
**Definition 3 (Voluntary participation condition)** In the mechanism *M*, any agent *i*, as long as it honestly bid, it cannot get negative profits, then this mechanism to meet the voluntary participation condition.

In a mechanism, a participant is called an agent, there are usually *n* agents, each agent *i* (*i* = 1,2,…,*n*) has some private information which is known as the type of the agent or called the true value *t_i_*, the private value is only known by agent, and is confidential for the other agents. For example, the type of an agent *t_i_* can be the cost executing an assigned task. *v_i_* (*t_i_, λ*) is the value function of an agent *i*, said the cost of performing a task. *p_i_* (·) is the payment function for agent performing a task. *u_i_* (·) is the utility function of an agent, that *u_i_* (·) = *p_i_* (·) − *v_i_* (*t_i_, λ*). *t_i_* is the true value, and *t̃_i_* is the implementation value [55]. Define vector *t* = (*t_1_,t_2_,…,t_n_*), *b* = (*b_1_,b_2_,…,b_n_*), *t̃* = *t̃*_1_, *t̃*_2_, ⋯, *t̃_n_*), and the output vector *λ* (**b**) = (*λ*_1_ (**b**), *λ*_2_ (**b**),…, *λ*_n_ (**b**)). Vector **b***_-i_* does not include the value *b_i_*, that is **b** = (**b**_−*i*_, *b_i_*). In a mechanism design, strategy proof condition will make all participants report their true value, and voluntary participation condition can ensure that all participants are willing to participate.

### 2.5. Repeated Game Theory

Repeated game theory is an extensive form game theory [12], a player has to take into account the impact of its current action on the future actions of others. Agah and Das [100] studied a repeated game formulation between malicious sensor nodes and an intrusion detector in the case of preventing passive Denial of Services (DoS) attacks at routing layer in WSN. Yang *et al.* [101] tackled the problem of dropping packets attacks in WSN, and modeled the interactions among sensor nodes as a repeated game. Xidong *et al.* [102] applied game theoretic dynamic power management policy for distributed WSN using repeated games. Yan *et al.* [103] defined a Contention Window Select Game (CWSG) in which each sensor node selects its own contention window to control the access probability, and proved the unique existence of NE in the CSWG. A penalizing mechanism based on repeated game theory to prevent the non-cooperative selfish behavior of decreasing the contention window without permission was proposed. Liu *et al.* [104] suggested a repeated game theoretic model. The model is based on cooperative packet forwarding under the conditions of selfish and rational nodes for improving energy efficiency and sensor networks payoff. The authors also formulated a payoff function on path reliability and energy consumption. Using the punishment mechanism, this repeated game model can propel a NE and decrease the defection possibility of selfish nodes. Pandana *et al.* [105] developed a self-learning repeated-game framework to enforce and learn the cooperation among the greedy nodes in packet forwarding. Zhou *et al.* [106] proposed a repeated game framework with a punishment mechanism to optimize packet forwarding probabilities by detecting, responding and punishing the nodes having selfish behaviors.

## 3. Applications of Game Theory in WSN

Game theory has many applications in WSN. Generally, we will consider two or more applications as an integrated domain rather than reviewing them separately. For example, in literature [77], data collecting, energy consumption, saving, and routing protocol design are considered jointly. The most significant roles of game theory in the design of WSN are summarized in Table 3.

### 3.1. Game Theory for Wireless Network Management in WSN

**Figure 3.**
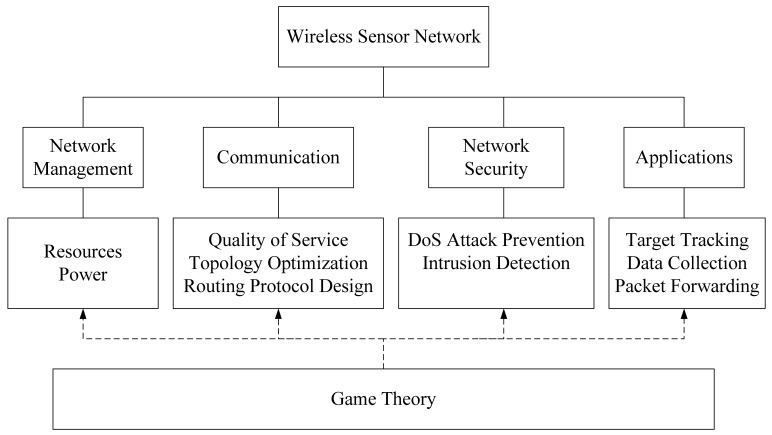
An illustration of the relation between WSN and game theory.

The design of a wireless network and optimization of its performance is a non-trivial and complicated process. The WSN has to fulfill straight requirements imposed from a set of operation goals. Game theory thus plays a supportive and critical role in designing and operating a WSN. Figure 3 illustrates the relationship between various WSN elements and the way that game theory is employed.

Representative requirements in a WSN include network management which is responsible for making the most economical use of power resources such that WSN can be put into operation for an extended period of time. A fundamental need for the WSN is to communicate with a centralized or base station that sensed data is fused and analyzed. In order to derive an effective WSN, one needs to consider the quality of service (QoS) specified, the topology used in the network architecture and how data is dispatched according to some preferred routing protocol. When the WSN is put into operation, it is always susceptible that the network may be attacked by hackers where data could be intercepted and retrieved illegally. To this end, the WSN has to be designed for deny of service (DoS) prevention and the incorporation of intrusion detection capability. With the desire to enlarge the application domain of WSN, attentions are also directed toward target tracking and data collection in the WSN design.

#### 3.1.1. Resource Management—Spectrum Allocation and Bandwidth Allocation

It is a salient and desirable feature that wireless sensors are deployed in a large number and with a high degree of redundancy. The sensors are therefore mostly manufactured with small size, light weight. Hence the resources, such as power, computation and communication capabilities, that can be embedded onboard the sensor are always limited. Resource management is thus an important consideration while designing a WSN.

Impulse Radio-Ultra WideBand (IR-UWB) communication is a strong candidate for short-range biomedical wireless sensor networks (BWSN). Moussavinik *et al.* [35,36] proposed a multiband/multiuser IR-UWB scheme, which is robust against unknown NarrowBand Interference, by deploying multiband signaling with Forward Error Correction coding and subband scheduling. They defined the problem with an *n*-transmitter Jamming game and find a NE for such a game. Furthermore, they cast the problem of finding the fair sub-band schedule into a multi-objective integer programming problem, and planed to solve it using NBS. Ma *et al.* [130] solved problems such as the optimal coverage of WSN or channel time-sharing in wireless communications inspired by the territory behaviors and mating systems of dragonflies.

Fu and Kozat [2] developed a virtualization framework to separate the network operator (NO) who focuses on wireless resource management and service providers (SP) who targets distinct objectives with different constraints. Within the proposed framework, the authors modeled the interactions among SPs and NO as a stochastic game, each stage of which is played by SPs and is regulated by the NO through the Vickrey-Clarke-Groves (VCG) mechanism. Due to the strong coupling between the future decisions of SPs and lack of global information at each SP, the stochastic game is notoriously hard. They introduced conjectural prices to represent the future congestion levels the end users potentially will experience, via which the future interactions between SPs are decoupled. Then, the policy to play the dynamic rate allocation game becomes selecting the conjectural prices and announcing a strategic value function at each time. They proved that there exists one NE in the conjectural prices and, given the conjectural prices, the SPs have to truthfully reveal their own value function. They further proved that this NE results in efficient rate allocation in virtualized wireless network. Huang *et al.* [131] considered a Markovian dynamical game theoretic setting for distributed transmission control in a WSN. The available spectrum bandwidth is modeled as a Markov chain. A distributed algorithm named correlated Q-learning algorithm was proposed to obtain the correlated equilibrium policies of the system. The problem of sequential estimation and lifetime maximization in a WSN was formulated in a cooperative game theoretic framework in [132,133], which allow the issue of fair resource allocation for sequential estimation task at the “fusion center” in a WSN as a solution of a cooperative game.

#### 3.1.2. Power Management—Energy Saving and Power Control

In WSN, energy is a limited resource and must be used judiciously. Currently, the energy problem remains one of the major obstacles somehow preventing the complete exploitation of WSN technology. Energy saving and power control strategies should be devised at sensor nodes as well as in the network to prolong the network lifetime. In practice, the Game-theoretical Total Link (GTL) algorithm which sets each node's energy range is usually better than Critical Transmitting Range (CTR) in energy saving according to the topological changing, as shown by Zhang *et al.* [122]. Kyung *et al.* [123] considered a MAC scheme based on *p*-persistence slotted ALOHA. To determine the value of the attempt probability *p*, the authors constructed the *p*-persistence slotted ALOHA as a simple non-cooperative game which involves generalized payoffs reflecting energy saving and throughput. They derived an NE in a closed form. Zhang *et al.* [27] investigated a cooperative communication scheme for WSN. They modeled the power allocation problem as a two-person bargaining game, and used the Nash Bargaining Solution (NBS) [27,35,36,42–47] to achieve a win-win strategy.

A distributed optimization framework using GT was used to analyze the power-allocation and rate allocation in WSNs [134]. Na *et al.* [113] proposed a distributed power control algorithm based on game theory for WSN, and its objectives are formulated toward reducing power consumption, decreasing overhead and increasing network lifetime. The simulation shows that this power control algorithm converges to a NE when decisions are updated according to a better response dynamic. The heterogeneous types of sensors are viewed as intelligent agents interacting with each other locally in a WSN. Sensors in this network interact with each other either in a cooperative way to form coalitions or in a non-cooperative way to deal with conflicts. Ren *et al.* [114] addressed non-cooperative game and cooperative game in a distributed power control algorithm to ensure reliability as well as power efficiency by proposing power allocation algorithms for network formation resulting from both selfish nodes and cooperative nodes. The contribution in [115] has a mechanism for obtaining smooth non-monotonic reaction curves, in contrast to sharp cut-offs with increasing interference that are characteristic of admission control. They [115] provided sufficient conditions for a unique NE under non-monotonic power control (NMPC) algorithm, and view this solution as edan attractive alternative to pricing in wireless networks formed by cooperative nodes.

Alpcan *et al.* [116] presented a game-theoretic treatment of distributed power control in CDMA wireless systems using outage probabilities and proved that the non-cooperative power control game considered admits a unique NE for uniformly strictly convex pricing functions and under some technical assumptions on the SIR threshold levels. Chen *et al.* [117] proposed a power control mechanism based on game theory to reduce power consumption of a WSN. The issue of restrictive energy in WSN was abstractly analyzed, and then the distributed self-adaptive power control algorithm was studied by analyzing the mapping between WSN and the game. The algorithm can reduce power consumption, decrease overhead and increase network lifetime, and converge to a NE when decisions are updated according to a better dynamic response and ensure nice fairness for network. Kannan *et al.* [119] presented a novel formulation of the problem of energy misbehavior and develop an analytical framework for quantifying its impact on other nodes. They formulated two versions of the power control problem for WSN with latency constraints arising from duty cycle allocations. In the first version, strategic power optimization, nodes are modeled as rational agents in a power game, who strategically adjust their powers to minimize their own energy. In the other version, joint power optimization, sensor nodes adjust their transmission powers to minimize the aggregate energy expenditure. Khayatian *et al.* [14] introduced novel concepts for relay selection schemes based on coalitional game theory which establishes the dynamic formation of coalitions in multi-user wireless networks and present a power allocation for wireless data transmission among coalition based on a non-cooperative game. Through this games theoretical framework, source nodes are able to self organize and form a stable coalition, and set their transmission powers in a completely distributed manner.

Niyato *et al.* [28] presented an optimal energy management policy for a solar-powered sensor node that uses a sleep and wakeup strategy for energy conservation. The problem of determining sleep and wakeup probabilities is formulated as a bargaining game. Moreover, NE is used as the solution of this game. Huang *et al.* [111] presented a pricing-based distributed power control scheme to compensate interference in sensor networks, inspired by the non-cooperative power control game studied in [112]. The misbehavior of terminals would create a social-dilemma where terminals exhibit uncertainty about the cooperative behavior of other terminals in the network. Cooperation in social-dilemma is characterized by a sub-optimal NE where wireless terminals opt out of cooperation. To maintain a socially optimal cooperation, a mechanism should be established to detect and mitigate effects of mis-behavior.

### 3.2. Game Theory for Wireless Communication in WSN

Wireless communication is a fundamental and critical component in a WSN. The effectiveness of the communication capability has to be addressed in every instances of deploying a WSN for operation. In addition to the power consumed in transmitting and receiving a radio signal, how the data is dispatched and routed within the network also crucially affects the WSN communication capacity.

#### 3.2.1. Wireless Communication

Dehnie and Memon [135] analyzed effects of misbehavior based on game theoretic approaches to design a mechanism which would enable wireless terminals to select reliable partners in the presence of uncertainty. The reputation mechanism introduced would lead to a perfect Bayesian equilibrium. Fu-Yun *et al.* [58] used a Bayesian game-theoretic approach to model transmission control in energy-harvesting WSN. In this problem, the energy state of an energy-harvesting sensor varies more dramatically with time as compared to traditional battery-powered sensors. Each sensor decides its transmission strategy according to its belief of its opponents' energy states. There exists a Bayesian Nash Equilibrium (BNE) where a sensor with energy higher than its energy threshold. Authors of literature [58] showed how each sensor determines its threshold to maximize its utility function. They demonstrated via simulations that the performance of the Bayesian game model is close to that of a perfect-information game where energy states are common information to all sensors. In addition, since the Bayesian game has the advantage of requiring less information exchange overhead, it seems to be more feasible to implement than the perfect-information game.

Kondi and Bentley [45] proposed a game-theory-based cross-layer optimization scheme for wireless Direct Sequence Code Division Multiple Access (DS-CDMA) visual sensor networks. The scheme uses Nash Bargaining Solution (NBS) which assumes that nodes negotiate with the help of a centralized control unit on how to allocate resources. The NBS takes into account the video quality, and each node could achieve without making an agreement. The cross-layer optimization scheme determines source coding rate, channel coding rate, and transmission power for each node. A virtual multiple input multiple output wireless sensor network (VMIMO-WSN) communication architecture is considered and the power control of sensor nodes based on the approach of game theory was formulated in [120]. The game was categorized as an incomplete information game, in which the nodes do not have complete information about the strategies taken by other nodes. For virtual multiple input multiple output wireless sensor network architecture considered the NE is used to decide the optimal power level at which a node needs to transmit and to maximize its utility. Valli *et al.* [121] proposed a power control solution for WSN considering MIDRS code in the analytical setting of a game theoretic approach. The game is formulated as a utility maximizing distributed power control game while considering the pricing function and the existence of NE is premeditated.

#### 3.2.2. QoS Control

Quality of Service (QoS) seems an overused term with various meanings and perspectives, but anyway QoS control is of paramount importance in WSN. The network is required to provide the QoS services while maximizing network resource utilization. To achieve this, the network needs to analyze the application requirements and deploy various network QoS mechanisms. Ayers and Yao [24] proposed a QoS control algorithm which is referred to as Gureen Game. Gureen Game not only improves the Gur Game for QoS control but also significantly addresses the power consumption weakness of the original Gur Game based QoS control for senor networks. Zhou and Mu [25] related the QoS with the node density and its control strategies were given for rectangular, circular, and various elliptical areas and designs for a dynamic adjusting mechanism based on the Gur Game algorithm. Cui *et al.* [118] presented a power control game theoretic approach for wireless multimedia sensor networks (WMSNs) by studying the effect of transmission power on QoS and energy efficiency. Danak *et al.* [136] proposed a novel management idea for positioning of sensor agents based on the concepts of Stackelberg games to enhance both the QoS and the network's robustness. Under a circumstance of intelligent transportation systems, a modified Gur game strategy based on localized information was given to determine the optimal alive node number in a focused area [1]. In the strategy, all nodes use local information to modify their finite state automaton, and the base station receives the QoS feedback and gives the dynamic density domination information.

To provide a set of measurable service attributes to the end users, Ke *et al.* [137] guarantee to measure the qualitative performance of QoS routing model, such as bandwidth, delay and delay jitter. A path that satisfies with the QoS was found by the Directed Diffusion (DD) algorithm, and an incomplete information game routing model was presented under this condition. A notion using routing game and ant colony algorithm to solve the QoS routing problem of wireless multimedia sensor networks was proposed in [138]. A mixed strategy routing game model was also proposed, and proves that the routing game has NE. A queuing analytical model was presented to investigate the performances of different sleep and wakeup strategies in a solar-powered wireless sensor/mesh network where a solar cell is used to charge the battery in a sensor/mesh node in literature [139]. The presented queuing model, along with the game-theoretic formulation, would be useful for the design and optimization of energy-efficient protocols for solar-powered wireless sensor/mesh networks under QoS constraints.

The multi-constrained QoS-based routing problem of wireless multimedia sensor networks is an NP hard problem. Genetic algorithms (GAs) have been used to handle these NP hard problems in wireless networks. Because the crossover probability is a key factor of GAs' action and performance, and affects the convergence of GAs, and the selection of crossover probability is very difficult, Zongwu *et al.* [140] proposed a novel method - crossover game instead of probability crossover. The crossover game in routing problems is based the principle that each node has restricted energy, and each node trend to get maximal whole network benefit but pay out minimum cost. The players of crossover game are individual routes. The individual would perform crossover operator if it is NE in the crossover game.

Body Sensor Networks (BSNs) provide continuous health monitoring and analysis of physiological parameters. A high degree of QoS for BSN is desperately required. Inter-user interference is introduced by the simultaneous communication of BSNs congregating in the same area. Wu *et al.* [141] proposed a decentralized inter-user interference suppression algorithm for BSN, called the Decentralized inter-user Interference Suppression in body sensor networks with non-cooperative Game (DISG).

#### 3.2.3. Topology Optimization

WSN has been used in many areas such as military sensing, environmental health monitoring, and home area security surveillance. Routing algorithm and topology control are extremely important in WSN to maintain a balance between throughput, delay and energy. Game theory has played an active role in this aspect. Zhao *et al.* [142,143] introduced an incompletely cooperative game-theoretic heuristic-based constraint optimization framework into WSN to acquire a balance. Varatharajan and Ercal-Ozkaya [3] considered a locally computable heuristic “recommendation algorithm” referring to as LocalHeur for convenience of incentive compatible topology control for selfish all-to-one routing which is modeled by a locally minimum cost forwarding game in the absence of complete global information for realistic scenarios. LocalHeur lies within the class of location based routing methods with particular similarity to the Nearest Forward Progress algorithm though from the perspective of game-theoretic reverse multicast routing. A game theoretic model presented in [37] yields decentralized optimization for joint topology control and power management. Global game equilibriums are iteratively reached by considering individual node degree, message delivery ratio and cost of increasing power. Researchers provide NEPow and BEPow schemes for implementations based on mathematical game analysis. Vuran and Akyildiz [144] designed a distributed, spatial Correlation-based Collaborative Medium Access Control (CC-MAC) protocol which based on the theoretical framework. The CC-MAC has two components: Event MAC (E-MAC) and Network MAC (N-MAC). As one of the most widely investigated topology control mechanisms of WSN, the clustering algorithm provides energy efficient communications by reducing transmission overhead and enhancing transmission reliability. Sarrafi *et al.* [126] developed a topology control algorithm for WSNs having a large scale structure and densely distributed nodes. The topology of the irrigation control sub-network is analyzed and a distributed two-tier routing (DTTR) protocol was proposed in literature [145]. The protocol includes intra-cluster and inter-cluster routing schemes. Game theory is introduced in the inter cluster multi-hop routing scheme. With the utilization of multistage finitely repeated games and the metric method based on link quality indication, the energy balance problem under multiple constraints is solved.

#### 3.2.4. Routing Protocol Designs

Routing protocol design is concerned with the efficiency that data, acquired by a sensor node, can be channeled through other nodes and either directly or indirectly reach the base station for analyze. Protocols used in WSN, on the other hand, have an integrated effect on the power consumed in radio transmission as well as the economical use of radio spectrum. Game theory has been satisfactorily employed in the design of routing protocols that it is able to account for difficulties in node behavior, energy balance, dynamic route allocation and many others. Game theory can also be applied in the control of *ad-hoc* network management, random access MAC and other communication architectures. Javidi and Aliahmadipour [107] had presented a review and comparison for typical representations of routing protocols applied game theory approaches for various wireless networks such as *ad-hoc* networks, mobile *ad hoc* networks and sensor networks that all of them lead to improve the network performance.

See-Kee and Seah [146] designed a generic protocol called Selfishness Resilient Resource Reservation protocol which applies game theory to achieve collusive networking behavior in WSN environment. Zhao *et al.* [147] modeled the problem of energy-efficient MAC protocols as a game-theoretic constraint optimization by introducing incompletely cooperative game theory which is based on the estimated game state. Ke *et al.* [148] presented a model of routing game for Wireless Multimedia Sensor Networks (WMSN). Authors discussed the equilibrium of the routing game, and gave a game routing algorithm for WMSN. Zheng [149] proposed a reliable routing model against selfish nodes and introduced this model into DSR routing as DSR-G, and the simulation result had shown that selfish nodes have less infections in DSR-G than DSR. In [68], a distributed energy balanced algorithm for data gathering and routing is proposed aiming to construct energy balanced routing trees in a network that contains heterogeneous nodes. Francesco *et al.* [150] proposed an enhanced access protocol which is basically based on modified version of Carrier Sense Multiple Access with Collision Avoidance (CSMA/CA) protocol. The scheme efficiency is proved resorting to game theoretic framework. The cooperative diversity protocols designed with the assumption that terminals always help each other in a socially efficient manner. However, the terminals may misbehave for selfish or for malicious intentions.

Kim *et al.* [151] proposed a game model to solve the MAC address assignment problem in sensor networks. Kim [152] developed a routing scheme based on the game theoretic model. By using the dynamic online approach, each sensor node can make routing decisions while ensuring good global properties. In addition, the proposed routing paradigm is realized in a distributed way without a central controller, which is more practical for real network operations. Krishnamurthy [70] formulated BNE conditions which a simple threshold strategy that is competitively optimal for each sensor, and proposed a scheme for decentralized threshold computation. Krishnamurthy [153] also considered two methodologies for decentralized sensor activation in WSNs for energy-efficient monitoring. Li *et al.* [154] abstracted the motivation of joint optimization of path reliability, energy consumption and lifetime factors as the rational tendency of intelligent sensors, and modeled the routing problem using a game theoretic paradigm. Liu *et al.* [155] proposed an efficient energy-management framework in WSNs and addressed the fundamental research challenge imposed by both the maintenance of the energy supply and the support of the quality-of-information (QoI) requirements. Mehta and Kwak [156] used the concept of incomplete cooperative game theory to model an energy efficient MAC protocol for WSNs. Valli and Dananjayan [157] analyzed a game theoretic model taking into consideration residual energy of nodes in a homogenous WSN considering random and square topology. A game theoretic method called the first price sealed auction game was introduced to control routing overhead in WSNs in literature [158]. The method divides all nodes in a WSN into several disjoint groups, and only one group is in the active mode at each time can save energy and prolong the lifetime of the whole WSN.

Lima and de Abreu [159] proposed and investigated a random access Medium Access Control (MAC)—Relay Selection Mechanism for cluster-based geographic routing in Multi-hop WSNs. The game-theoretical relay selection strategy relies on descending pricing auctions which is known as “Dutch Auctions”. Koltsidas and Pavlidou [160] provided a game theoretical modeling of clustering for *ad-hoc* and sensor networks. The analysis is based on a non-cooperative game approach where each sensor behaves selfishly in order to conserve its energy and maximize its lifespan. The authors formulate a clustering mechanism called Clustered Routing for Selfish Sensors (CROSS) which can be applied to sensor networks in practice. To balance energy consumption of sensor nodes and increase network lifetime and stability, a cooperative game theoretic model of clustering algorithms is provided for assigning feasible allocations of energy cost [125]. Wang *et al.* [161] proposed a trustworthy energy efficient routing algorithm (TEER) aiming to distribute energy consumption across sensors evenly as well as increase the path security in a hierarchical-cluster sensor network, and the cluster head is selected by using a game theoretic paradigm. Zhong and Cheng [162] introduced GT to routing algorithm and presented the Unequal Clustering Energy-Economical Routing (UCEER) algorithm for WSN which can balance nodes' energy consumption and prolong network lifetime. Yang *et al.* [163] proposed an optimal cluster-head algorithm to improve network performance.

To maximize the average coverage, Xin *et al.* [40] desired that the overlapped sensing area in each group to be minimized. To achieve this objective, authors formulated this group division and coverage maximizing problem into a game theory model and the desired solution in this model is a NE strategy profile. Then, a hill-climbing NE convergence idea applied into the coverage game and let each node converge to an approximate NE in a coherent local way. Zeydan *et al.* [124] proposed an efficient routing solution using a game theoretic framework for correlated data collection in WSNs. The Game-theoretical Complete Coverage (GCC) algorithm is used to ensure whole network coverage mainly through adjusting the covering range of nodes and controlling the network redundancy in literature [164]. A simplified game-theoretic constraint optimization scheme (G-ConOpt) was presented in literature [165], which is easy to be implemented in current WSNs. A simplified game-theoretic MAC protocol (G-MAC) was provided for WSNs in literature [166], by using an auto digressive back-off mechanism which is easy to be implemented. Zheng *et al.* [167] presented an energy-efficient Adaptive Clustering Hierarchy based on Game-theoretic Techniques (ACHGT) routing algorithm for WSNs to balance the energy consumption.

To balance energy consumption of sensor nodes and increase network lifetime and stability, a cooperative game theoretic model of clustering algorithms is provided for assigning feasible allocations of energy cost in literature [125]. Zeng *et al.* [168] proposed a Game Theoretic Energy Balance Routing (GTEBR) algorithm to avoid unevenly energy consuming in WSNs. Pandremmenou *et al.* [44] used the NBS in order to determine the transmission power and source and channel coding rate for each node to solve the problem of resource allocation for a Direct Sequence Code Division Multiple Access (DS-CDMA) wireless visual sensor network. The NBS assumes that the nodes using the help of a centralized control unit negotiate in order to jointly determine their transmission parameters. The transmission powers are allowed to take continuous values, whereas the source and channel coding rate combination can only assume discrete values. The resulting optimization problem is a mixed-integer optimization task and is solved using Particle Swarm Optimization (PSO). Zhu and Jian [169] proposed an efficient algorithm for computing the optimal strategies for jamming attack and network defense. Yan *et al.* [170] introduced a novel pure distributed method for solving K-Cover problem based on non-cooperative game theory.

### 3.3. Game Theory for WSN Security

Due to the limited capabilities of sensor nodes in terms of computation, communication, and energy, providing security to WSN has increasingly become one of the most interesting areas of research in recent years. WSN security is a primarily important and critical issue before WSN can be widely used. There usually exist two mechanisms of intrusion prevention and detection in WSN security. GT provides a mathematical method for analyzing and modeling WSN security problems for it considers scenarios where multiple players with contradictory objectives compete with each other. Shen *et al.* [6] had proposed a taxonomy which divides current existing typical game theory approaches for WSN security into four categories: preventing DoS attacks, intrusion detection, strengthening security, and coexistence with malicious sensor nodes. They pointed out some future research areas for ensuring WSN security based on game theory, including Base Station credibility, IDS efficiency, WSN mobility, WSN QoS, real-world applicability, energy consumption, sensor nodes learning, expanding game theory applications, and different games.

#### 3.3.1. Preventing DoS Attacks

The game types for preventing DoS attacks include non-cooperative game [171–173], cooperative game [174], and repeated game [100,101]. The jamming and anti-jamming issues are modeled as a zero-sum stochastic game in literature [175] to defend DoS attack. In this game, the actions of the sensor and jammer are dependent on the current system state. A quadratic function is used as the payoff function, thus facilitating the LQG control of the power system. The NE of the game is analyzed, including the existence and the corresponding computation. Numerical simulations are carried out for a seven-dimensional linear system of power grid and demonstrate the increase of reward when proper anti-jamming actions are taken. Dong *et al.* [176] established an attacking-defending gaming model which can detect active DoS attacks effectively, where the strategy space and payoff matrix are given to both the IDS and the malicious nodes.

#### 3.3.2. Intrusion Detection

The game types for intrusion detection include non-cooperative game [177–181] and Markov game. Reddy [179] discussed currently available intrusion detection techniques; attack models using game theory, and then propose a framework to detect malicious nodes using zero sum game approach for nodes in the forward data path. Mohi *et al.* [39] modeled the interaction of nodes in WSN and IDS as a Bayesian game formulation and use this idea to make a secure routing protocol. By this approach nodes are motivated to act rationally and gain reputation, and IDS can perform better by using the history of the game. This game approaches to NE and leads to a defense strategy for the network. Reddy and Srivathsan [33] developed a framework to detect malicious nodes using Zero-Sum game approach and selective node acknowledgements in the forward data path. A game theory-based scheme is introduced for finding out the vulnerable areas in WSN [174,177]. Non-cooperative game theory-based schemes could assist detection schemes in improving their performance as well as efficiency though they are not concerned with detection directly [182]. Qiu *et al.* [15] proposed an active defense model for WSN where nodes have limited rationality of evolutionary learning ability based on evolutionary game theory. The nodes can achieve the most effective defense by adjusting their defensive strategies active and dynamic. Ma and Krings [16–19] envisioned a WSN as an entity analogous to a biological population with individual nodes mapping to individual organisms and the network architecture mapping to the biological population. The interactions between individuals, either insects or WSN sensors, can be captured with evolutionary game theory models, in which individuals are the players and reliability are the fitness (payoff) of the players. The evolutionary stable strategy (ESS) forms the basis of network survivability. Ma and Krings [20] introduce a layered modeling architecture consisting of dynamic hybrid fault modeling and extend evolutionary game theory for reliability, survivability, and fault tolerance analyses. The architecture extends traditional hybrid fault models and their relevant constraints in the agreement algorithms with survival analysis, and evolutionary game theory. Selfish nodes refuse to forward packets for other nodes in order to save energy which causes the network to malfunction. At the same time, some nodes may be malicious, whose aims are to damage the network. In [67], Jing and Ruiying analyzed the cooperation stimulation and security in self-organized WSN under a game theoretic framework.

### 3.4. Game Theory in Applications of WSN

#### 3.4.1. Target Tracking

A method for target tracking based on multi-agent and game theory was proposed in [109,110]. When a target appears in the sensing field of WSN, sensor nodes begin to form coalition dynamically and then they start to negotiate with game theory. Coalition is formed to track it with the target moving. Utilizing multi-agent method and game theory in WSN enables nodes to perform tasks coordinately to achieve some desired objectives.

#### 3.4.2. Data Collection

The collection of sensor data from a site is one of the most important issues in WSN. A good packet forwarding strategy has to be developed for balancing and selecting transmission packets. In data collection, it is important to ensure that all sources have equal access to the network bandwidth so that the sink receives a set of data with complete coverage in the site. Zhao and Shi [29] developed a Packet Forwarding algorithm based on the Dynamic Bayesian Game named PFDBG for WSN. The adjacent nodes in WSN use the Bayesian amendment method to reasonably calculate and predict the opponent's energy level based on an evaluation of its historical actions to maximize the expected utility function. Seredynski and Bouvry [127] introduced a Prisoner's Dilemma-based model of packet forwarding and next using an evolutionary game-theoretical approach. They demonstrated that cooperation is very likely to be developed on the basis of conditionally cooperative strategies similar to the TIT-FOR-TAT strategy. Xie *et al.* [183] proposed a bargain-based mechanism to encourage cooperative message trading among selfish nodes to maximize their rewards. Shi *et al.* [184] proposed an approach for MAC design with prioritized event reporting in WSNs based on a game theoretic framework. The MAC priority classes are adaptively assigned based on accuracy of location information for individual sensor nodes, and on data correlation structure. The MAC solution makes event reporting more accurate and event localizing faster. Lin *et al.* [185] proposed a differential game model to stimulate forwarding based on the payoff value of the differential game called forwarding contribution by which selfish avoidance clustering in WSNs was considered. Lu [186] used game theory to analyze cooperative mechanisms and derive incentive strategies enforcing cooperation in forwarding.

#### 3.4.3. Packet Forwarding

In WSNs, selfish nodes refuse to forward packets for other nodes in order to save energy which causes the network to enter into a faulty state. At the same time, some nodes may be malicious, whose aims are to damage the network. Chen and Du [67] analyzed the cooperation stimulation and security in self-organized wireless networks under a game theoretic framework. The fault tolerance and security problem is modeled as a non-cooperative game in which each player maximizes its own utility function. The results showed that players can obtain the biggest payoff if they tell the truth and obey the cooperation strategy. Dai *et al.* [41] adopted the viewpoints and methods in game theory to solve the packet forwarding problem in WSNs and proposed an algorithm named Pareto Optimal Utility based Packet Forwarding (POUPF) which is able to set a NE and to obtain the Pareto optimal utility for each node. Dehnie *et al.* [187] formulated the interaction between sensors and monitoring nodes as a dynamic game with incomplete information, which provides a platform for designing reputation based data fusion. Huang and Liu [188] studied the tradeoff between data transmission delay and overall network energy efficiency, and developed a game-theoretic model of real-time reliable aggregation mechanism for WSNs. Jia *et al.* [128] focused on the packet forwarding problem in WSNs and provided a solution with the method of game theory.

A common aggregation model was proposed in [23], which is independent of the specific application environments, but based on the evolutionary game theory called evolutionary game-based data aggregation model (EGDAM) in WSNs. EGDAM consists of formal definition, functional model and a general process is defined to map the competition and cooperation in aggregation procedure into games, and well-avoid perfect rationality. Guided by the model, an evolutionary game-based adaptive weighting algorithm named EGWDA was provided for the pixel-level data aggregation with homogeneous sensors. Reasonable sensors weight distribution can be achieved during the aggregation process in WSNs. Fan *et al.* [189] proposed a scheme to reach shorter multicast delay, better energy utilizing efficiency and higher efficiency of data transferring for Sensor Grid. Zeydan *et al.* [190] proposed an efficient bottleneck throughput maximizing routing framework for correlated data gathering in WSN. In [144], [82] and [191], the estimation reliability in the mean square error (MSE) criterion was defined, and it is between a source node in the sensor field and its estimation at a sink node. Kazemeyni *et al.* [4] considered the cooperation of nodes in data transmission in terms of a group, since the major consumer of power is the data transmission process. A mobile node may move to a location, in which it is desirable for the node to join a group. They proposed an algorithm for nodes to choose the best group in their signal range, using coalitional game theory to determine what is beneficial in terms of power consumption.

### 3.5. Other Uses of Game Theory in WSN

#### 3.5.1. Evolutionary and Soft Computing

Researchers also attempted to use GA for WSN in other aspects. For instance, Yang *et al.* [192] proposed Game Optimization CSMA (GO/CSMA) to optimize CSMA mechanism. He and Gui [74] used GT model to realize NE and node set's rational division. Li *et al.* [193] first proposed an autonomic management framework (ASGrid) to smooth the integration of WSN and grid systems. Li *et al.* [194] developed an agent-based negotiable game theoretic sensor management (ANGSm) approach incorporating sub-game NE into negotiation. Holes, hindering the information flow in WSN, happen when one or more sensors are not functioning or few sensors are missing during deployment in any designated area. Reddy [195] presented the potential game model for detecting holes in WSN. A coverage optimization problem for mobile visual sensor networks as a repeated multi-player game was formulated. A synchronous distributed learning algorithm where each sensor only remembers its own utility values and actions played during the last two time steps was presented in literatures [196,197]. Abdelkader *et al.* [198] proposed a technique to implement self-organization strategies for WSN. The process of iterating the computation of the Voronoi diagram generated by the sensors has a maximum fixed point which corresponds to the NE of a strategic game where sensor nodes move iteratively to the centers of the Voronoi cells. This fixed-point represents the best deployment that can be achieved from the coverage point of view. Altman *et al.* [199] applied evolutionary games to non-cooperative networks containing a large number of individual non-cooperative terminals or sensors and proposed some guidelines for designing a framework that supports evolution of protocols. A pricing and payment technique was presented in [200] to obtain an optimal path in a WSN by considering reliability, energy and traffic load. The proposed algorithm is able to find a path which improves network lifetime, load distribution and path reliability.

A robust design using fuzzy interpolation in the viewpoint of GT was given in [201]. The fuzzy approximation error for the nonlinear dynamics of multiple sources is incorporated into the design. Closas *et al.* [202] proposed a distributed algorithm which is based on the non-cooperative game principle. Crosby and Pissinou [22] introduced the concept of multi-class wireless sensor networks where each class is governed by a different authority to study the evolution of cooperation in static and mobile multi-class WSN using evolutionary game theory. The proposed algorithm, called Patient Grim Strategy, provides an NE solution to the game theoretic problem of cooperation in multi-class static wireless sensor networks. Danak *et al.* [203] introduced a novel game theoretical approach to the sensor management problem considering each node as an intelligent agent and using the concepts of the theory of auctions.

Li *et al.* [204] proposed a Distributed Source-Relay Assignment (DSRA) algorithm for competitive price adjustment. A two-side market game approach is employed to jointly consider the benefits of all sources and relays. The equilibrium concept in such games is called the core. The outcomes in the core of the game cannot be improved upon by any subset of players. These outcomes correspond exactly to the price-lists that competitively balance the benefits of all sources and relays. The core of the game is defined as discrete core when the price assumes only discrete values. A patient is equipped with health parameter transducers which are connected to a WSN. Moreover, the WSN will perform some computations and will raise an alarm when some diseases are suspected [205]. A distributed algorithm, *i.e.*, the decision may be done by any mote dealing with the disease detection, is proposed to solve the problem. Estiri and Khademzadeh [206] presented a signaling game-theoretic model to analyze intrusion detection in WSN. They used a signaling game to model the interactions among nodes of a WSN. They viewed the interaction between an attacker and an individual node as a Bayesian game with incomplete information, and construct models for such a game. Estiri and Khademzadeh [34] proposed a game-theoretical based method to fortify an intrusion detection system (IDS) in WSN. They formulated the attack-defense problem as a non-cooperative, two-player, non-zero-sum game between an attacker and a WSN. They proposed a repeated game model of dropping packets which is attack-proof against dropping packets attacks based on such an assumption that sensor nodes are rational. The model prevents malicious nodes from attacking by establishing sub-game perfect periodic collusion-resistant punishment mechanism, and impels sensor networks to reach a cooperative NE.

#### 3.5.2. Others

Felegyhazi *et al.* [207] introduced the concept of multi-domain sensor networks to a WSN, it contains a game-theoretic model to investigate the impact of cooperation, and shows conditions for which cooperation is the best strategy. Nayer and Ali [26] used Gur Game in order to achieve the optimal assignment of active sensors while maximizing the number of regions covered by sensor nodes. They used a dynamic clustering algorithm that employs the concept of connected dominating sets. The proposed algorithm addresses this problem by playing the Gur Game among the cluster nodes. They also further developed the earlier ants algorithm and genetic algorithm to take into consideration node addition and deletion. Karnik *et al.* [208] formulated two approaches for optimization of computing rates. The first is a team problem for maximizing the minimum communication throughput of sensors, and the second is a game problem in which cost for each sensor is a measure of its communication time with its neighbors. The game formulation not only leads to an explicit characterization of the NE but also to a simple iterative scheme by which sensors can learn the equilibrium attempt probabilities using only the estimates of transmission and reception times from their local measurements. Krishnamurthy *et al.* [209] presented decentralized adaptive filtering algorithms for sensor activation control in an unattended ground sensor network (UGSN) comprised of ZigBee-enabled nodes. Krishnamurthy and Hanh [210] considered finite-size slotted ALOHA sensor networks with multiple packet reception capability and selfish sensors. The problem is formulated as a finite player finite action, non-cooperative stochastic game where each sensor is a selfish but rational player.

Instead of the Blackwell approachability method used in previous works, Krishnamurthy *et al.* [211] gave an ordinary differential equation formulation with a Lyapunov function to prove convergence to a correlated equilibrium. Kwanghyun *et al.* [129] dealt with a scheduling algorithm to fulfill a qualified end-to-end video service in terms of fairness and energy efficiency over multi-hop wireless sensor networks. In [212], on the basis of comprehensive consideration of game theory and the IEEE 802.11 Distributed Coordination Function (DCF), for the characteristics of the WSN, the authors put the channel competitive process between nodes molded into an incompletely information dynamic game. Then improve DCF by the NE game strategy. The simulation results show that the proposed model and strategy can obtain higher efficiency than the 802.11 DCF and improve many performances of the WSN.

Maskery and Krishnamurthy [213–215] described a decentralized game theoretic adaptive algorithm and learning-based activation algorithm for a ZigBee-enabled unattended ground sensor network. See-Kee and Seah [216] applied GT to achieve collusive networking behavior in WSNs. Shah and Kumar [60] proposed, COllective INtelligence (COIN), a macro-learning paradigm that aims to specifically address the problem of designing utility functions for individual agents in order to achieve higher system wide utility. They extended the method of distributed independent reinforcement learning, by combining it with COIN based macro-learning paradigm to steer the system towards global optimization, and improve performance with minimal communication overheads. Zichong *et al.* [32] presented a two-encoder scheme which imitates the ping-pong game and has a successive approximation structure. This ping-pong distributed coding idea can be extended to the multiple encoder case and provides the theoretical foundation for a new class of distributed image coding method in wireless scenarios. Czarlinska *et al.* [217–219] employed the game-theoretic analysis in wireless visual sensor network (WVSN) and wireless sensor actuator networks (WSANs) which are concerned about security. Current advances in wireless sensor networks have contributed to the development of multi-hop wireless body sensor networks (WBSNs). Pal *et al.* [43] model the problem of reliability optimization in multi-hop WBSNs as a cooperative game which inherently induces a distributed mechanism that optimizes the reliability of network operations in multi-hop WBSN supporting concurrent applications. The NBS optimizes the overall system reliability. WSANs combine sensors and actuators interconnected by wireless networks in order to perform distributed sensing and action tasks. The model and simulation method as well as the framework which was proposed in [220] are illustrated on a Pursuit Evasion Game. Ren *et al.* [221–223] proposed a bio-effects metric to evaluate the adverse biological effects using game theory. Ma *et al.* [21] solved the problems such as the optimal coverage of WSN or channel time-sharing in wireless communications inspired by the dragonfly-behavior, using mathematical modeling tools such as evolutionary game theory and rendezvous search game theory are suggested to transfer biological inspirations to engineering designs.

## 4. Future Research Directions

Although GT has been employed as efficient approaches for WSN, some problems still exist in its application in this area. Researchers are exerting efforts not only in simple energy saving and topology control applications, but also in improving the methods mainly in the following aspects.

### 4.1. Moving Target Tracking

Moving targets can be considered as intelligent agents and this characteristic often increases the tracking difficulty. On the other hand, sensor nodes only have limited wireless communication capability. Therefore, there is a need to find a way to cooperate efficiently in target estimation.

Gu [108] applied a zero-sum game approach to the estimation of target position. The minimax filter is constructed to minimize the estimation error under the worst case noise, so it is robust to the adversary tracking difficulty imposed by moving targets. The work also developed a distributed version of the minimax filter with an improved performance. A sensor node communicates with its neighbors that are located in the range of wireless communication in the distributed minimax filter. A further investigation of a moving-target tracking problem with sensor networks was reported. Each sensor can observe the target by a sensor and estimate the target position by a processor. In this game, the tracking of the adversary moving target can use a strategy to maximize the estimation error. Moreover, how to track a moving target by taking the advantages of large spatial coverage, robustness, and cooperation is still challenging. Gu [108] formulated this target estimation problem as a zero-sum game and used a minimax filter to estimate the target position in addition to the distributed Kalman filter which had been one of the popular approaches for estimating the position of a moving target.

### 4.2. Scheduling

Sensing tasks should be allocated and processed among sensors in a minimum time, so that users can draw prompt and effective conclusions through analyzing the sensed data and can reduce power consumption to prolong the lifetime. Scheduling tasks in WSNs is one of the most challenging problems.

To remove the potentially selfish behavior of the sensors, a non-cooperative game algorithm for task scheduling in WSNs was proposed in [55]. In the proposed algorithm, according to the Divisible Load Theory, the load are divided into any-size parts, and they are distributed reasonably to every node from the sink on the basis of the processing and communication capability. By removing the performance degradation caused by communications interference and efficiency lost due to idle states, the decreased load completion time and the improved network resource utilization are achieved. Strategyproof mechanism can provide incentives to the sensors in order to force them to obey the prescribed algorithms, and to accurately report their parameters, it will lead to the demand for a more efficient task scheduling. A utility function relating the total task completion time, and tasks allocating scheme was then designed where the NE of the game algorithm was proved. The simulation results had shown that in a mechanism, selfish nodes can voluntarily report their true processing capability and try their best to participate in the mechanism, thereby, the total time for accomplishing the task is minimized and the energy-consuming of the nodes is balanced.

### 4.3. QoS Control

While a lot of contributions have been available on some important aspects of WSNs, supporting QoS in WSNs is still a largely unexplored issue. In mechanism design, each participating entity is often called an agent. In a mechanism, the participants can obtain the maximum benefit by reporting their parameters subject to a given algorithm. The mechanism of this kind is called a Strategyproof mechanism and also known as credible mechanism. In other words, in strategyproof mechanism, truth telling is a dominant strategy. A rational participant in order to maximize its interests will tell the truth. Strategyproof can well coordinate resource sharing among autonomous entities and collaboration to achieve the system's self-support and healthy development.

Mechanism design is the most important application of the non-cooperative game theory. Although the system can achieve NE by mechanism design, but often only a sub-optimal result is obtained. Pareto Optimality can be derived from cooperative game theory. The theory will help protect the overall performance of WSN. However, the cooperative game theory only considers the ideal computing model. This simplification not only ignores the corresponding QoS constraints, but also ignores the diversity of operations, resources, heterogeneous and dynamic environments. Therefore, it is necessary to consider QoS with reference to constrained cooperative game theory and focus on the following questions:

1. QoS constrained algorithmic mechanism design.
2. QoS constrained and group strategyproof distributed algorithmic mechanism design.
3. QoS constrained cooperative game theory.

Based on the ideal computing model for the specific application, one should study the mechanism designs of different characteristics by considering QoS constrained and in combination with the cooperative game theory.

### 4.4. Security

In WSNs under fully distributed conditions, there is no coordinator; participants need to communicate with each other to execute the mechanism, and to accomplish the task. In this way, network security will be the most important issue [224–227]. It is not only needed to design the appropriate network security protocol to protect communication security, but also to protect the participants from disclosing confidential information, such as private parameters, bids, and bidders logo. Wireless sensor network designs also need to consider the abnormal behavior of agents, the complexity of network communications and other considerations.

## 5. Conclusions

This paper has summarized the recent developments in Game Theory for Wireless Sensor Networks. GT has the capability to examine a larger amount of possible scenarios before performing the action. As a modeling tool, GT can make a decision process more sophisticated. The potential of applying GT to WSNs is prospective. Some researchers have already explored the game-theoretic approach to address WSNs design problems and have proposed some promising solutions. Here we have given the taxonomy of exiting approaches in order to provide to interested readers a global view of GT for WSNs. Representative contributions are listed to give a general overview of the state-of-the-art. Open challenges and future trends have been identified.

## Figures and Tables

**Table 1.** Typical GT methods in WSN.

	**Method**	**References**
(1)	Cooperative game theory	[8]
(2)	Non-cooperative game theory	[9,10]
(3)	Repeated game theory	[11,12]
(4)	Coalitional game theory	[4,7,13,14]
(5)	Evolutionary game theory (extended)	[15–23]
(6)	Gur game	[1,24–26]
(7)	Bargaining game	[27,28]
(8)	Dynamic Bayesian game	[29]
(9)	TU game (transferable-utility game)	[30]
(10)	NTU game (non-transferable-utility game)	[31]
(11)	Ping-pong game	[32]
(12)	Zero-Sum game and Non-Zero-Sum game	[33,34]
(13)	Jamming game	[35,36]

**Table 2.** Common Terminologies in GT.

	**Terminology**	**References**
(1)	Nash Equilibrium (NE)	[37–40]
(2)	Pareto Optimal	[41]
(3)	Nash Bargaining Solution	[27,35,36,42–47]
(4)	Shapley Value	[30,48]
(5)	Core	[49–51]
(6)	Mechanism Design (Computational)	[52–54]
(7)	Incentive compatible	[3]
(8)	Strategyproof Mechanism	[55]
(9)	Auction	[56]
(10)	Vickrey-Clarke-Groves (VCG) Mechanism	[57]
(11)	Utility Function	[7,29,55,58–68]
(12)	Bayesian Nash Equilibrium (BNE)	[37,58,69,70]

**Table 3.** The most significant roles of GT in WSN design.

	**Role**	**References**
(1)	Routing protocol design	[13,77,107]
(2)	Target tracking	[108–110]
(3)	Power control	[52,69,89,94,111–121]
(4)	Energy saving	[122,123]
(5)	Data collection	[77,124]
(6)	Topology control	[3,37,125,126]
(7)	Spectrum allocation	[90,96]
(8)	Bandwidth allocation	[42]
(9)	Packet forwarding	[29,41,104–106,127,128]
(10)	Task scheduling	[55,129]
(11)	Quality of Service (QoS) control	[24]
